# Ribosomal DNA Deletions Modulate Genome-Wide Gene Expression: “*rDNA*–Sensitive” Genes and Natural Variation

**DOI:** 10.1371/journal.pgen.1001376

**Published:** 2011-04-21

**Authors:** Silvana Paredes, Alan T. Branco, Daniel L. Hartl, Keith A. Maggert, Bernardo Lemos

**Affiliations:** 1 Department of Biology, Texas A&M University, College Station, Texas, United States of America; 2 Department of Organismic and Evolutionary Biology, Harvard University, Cambridge, Massachusetts, United States of America; Cornell University, United States of America

## Abstract

The ribosomal *rDNA* gene array is an epigenetically-regulated repeated gene locus. While *rDNA* copy number varies widely between and within species, the functional consequences of subtle copy number polymorphisms have been largely unknown. Deletions in the *Drosophila Y*-linked *rDNA* modifies heterochromatin-induced position effect variegation (PEV), but it has been unknown if the euchromatic component of the genome is affected by *rDNA* copy number. Polymorphisms of naturally occurring *Y* chromosomes affect both euchromatin and heterochromatin, although the elements responsible for these effects are unknown. Here we show that copy number of the *Y*-linked *rDNA* array is a source of genome-wide variation in gene expression. Induced deletions in the *rDNA* affect the expression of hundreds to thousands of euchromatic genes throughout the genome of males and females. Although the affected genes are not physically clustered, we observed functional enrichments for genes whose protein products are located in the mitochondria and are involved in electron transport. The affected genes significantly overlap with genes affected by natural polymorphisms on *Y* chromosomes, suggesting that polymorphic *rDNA* copy number is an important determinant of gene expression diversity in natural populations. Altogether, our results indicate that subtle changes to *rDNA* copy number between individuals may contribute to biologically relevant phenotypic variation.

## Author Summary

The repeated *rDNA* array gives rise to the nucleolus, which is one of the first described intracellular structures and is known to be involved in key cellular processes such as stress response, cell cycle regulation, RNA modification, and production of more than 90% of all cellular RNAs (the ribosomal RNAs). The *rDNA* exists in excess; and, although many copies are inactivated through epigenetic mechanisms, the biological significance of inactive copies has been a matter of debate. We present a system that allows for the identification of global gene expression effects stemming from differences in *rDNA* copy number. We have discovered that deletions in the *rDNA* locus result in the differential expression of hundreds to thousands of genes. This raises the expectation that important phenotypic variation affecting health and disease might be traced to polymorphic variation in *rDNA* copy number. Furthermore, the manifold effects of *rDNA* copy number indicate that considering polymorphisms in the *rDNA* might bring new light to studies of epigenetic inheritance and its contribution to the heritability of complex traits.

## Introduction

The ribosomal *rDNA* gene array is an epigenetically-regulated repeated locus responsible for producing over 90% of cellular RNAs. The large (*35S*) *rDNA* array gives origin to the Nucleolus Organizer Region (NOR) and the *35S* rRNA genes are present from fewer than 50 to more than 25,000 copies among eukaryotes [1]. However, even though copy number varies widely between and within species [2], [3], the functional consequences of copy number polymorphisms have been largely unknown, and often assumed to have negligible functional consequence. This is in part because redundant *rDNA* arrays are found in eukaryotic genomes. In humans, for example, *rDNA* arrays containing the *35S* rRNA genes are found on chromosomes 13, 14, 15, 21, and 22. Similarly, redundant *rDNA* genes within each array can compensate for copy number polymorphisms to maintain a sufficient supply of rRNAs [4]–[6]; indeed, only a fraction of the *rDNA* units in a particular array are transcribed at any given time [7], and flexibility in the transcriptional elongation rate allows for control of rRNA output in conditions of rapid cell division and growth. Hence, even though *rDNA* copy number and rRNA production is regulated by the cell, the relevance of supernumerary or inactive *rDNA* repeat units has remained elusive. Recent work has investigated whether inactive *rDNA* copies are necessary for genome stability [8].

In *Drosophila melanogaster*, *rDNA* arrays exist as a pair of functionally-redundant loci on the *X* and *Y* chromosomes [5], [6], which have further suggested that variable copy number in the *Y*-linked *rDNA* array might have little, if any, functional significance. Indeed, even though *X0* males are sterile due to loss of *Y*-linked fertility genes, they appear morphologically normal despite having no *Y*-linked *rDNA* [9]. Nevertheless, Paredes and Maggert [10] have recently shown that induced variation in the *Y*-linked *rDNA* copy number modifies heterochromatin-induced position effect variegation (PEV), and natural changes in *rDNA* copy number through development correlate with PEV. Taken together, these findings suggested that polymorphisms in *rDNA* copy number might be relevant to the maintenance of genome-wide chromatin structure.

Polymorphic naturally occurring *Y* chromosomes induce *Y*-linked Regulatory Variation (YRV), which affects the expression of autosomal and *X*-linked genes [11]. In *Drosophila melanogaster,* YRV is observed in males differing only in the origin of their *Y* chromosomes and it is manifested as the differential expression of hundreds of non-*Y*-linked genes [11]. The source of YRV cannot be simply ascribed to polymorphisms in protein-coding genes [12], [13], nor is it easily mapped to sub-regions of the *Y* chromosome because of the lack of recombination along the *Y*-chromosome and the difficulty in manipulating large segments of heterochromatin. The similar contributions of induced deletions in the *Y*-linked *rDNA* array and naturally occurring *Y* chromosome variation to PEV [10]–[13], suggested that naturally occurring *rDNA* copy number polymorphism might be a significant source of regulatory variation. The hypothesized association between Y-linked regulatory variation and *rDNA* copy number might provide a molecular framework for the contribution of the *Drosophila melanogaster Y* chromosome to adaptive phenotypic variation and fitness.

Here we tested the hypothesis that induced deletions in the *Y*-linked *rDNA* in an otherwise isogenic background might modulate variation in genome-wide gene expression. Our results indentified hundreds to thousands of genes whose expression is sensitive to deletions in the *rDNA*. Importantly, many of the induced changes in expression could be replicated in both male and female genotypes. Further, we show significant associations between genome-wide gene expression variation arising from *Y* chromosomes with targeted deletions in the *rDNA* loci and variation arising from naturally occurring polymorphic *Y* chromosomes, which harbor polymorphisms in *rDNA* copy number. This suggests that variation in *rDNA* copy number may be an underappreciated source of natural variation with important consequences to the modulation of gene expression across the genome.

## Results

To address the relevance of *rDNA* copy number to genome-wide gene expression we used *Y* chromosomes containing targeted reductions of *rDNA* copy number. These chromosomes are otherwise expected to remain structurally unchanged. Although it is possible that these chromosome differ in ways that we cannot detect, four lines of evidence suggest that alterations of the *rDNA* copy number are the sole difference between these chromosomes: (1) *I*-*Cre*I-mediated chromosome alterations were genetically and cytologically limited to the *rDNA* [14], (2) no other identifiable *I*-*Cre*I consensus site has been cloned from *Drosophila*, (3) the position effect variegation phenotype arising form these alterations were reverted concomitant with *rDNA* magnification [10], and (4) *Drosophila rDNA* is known to be comprised solely of *rDNA* and two retroelements [15], [16]. Although we cannot formally rule out the possibility that heretofore uncharacterized non-*rDNA* sequences are in the *rDNA* loci, for ease of discussion we consider that deletions in the *rDNA* are the major source of variation between these chromosomes.

**Figure 1:**
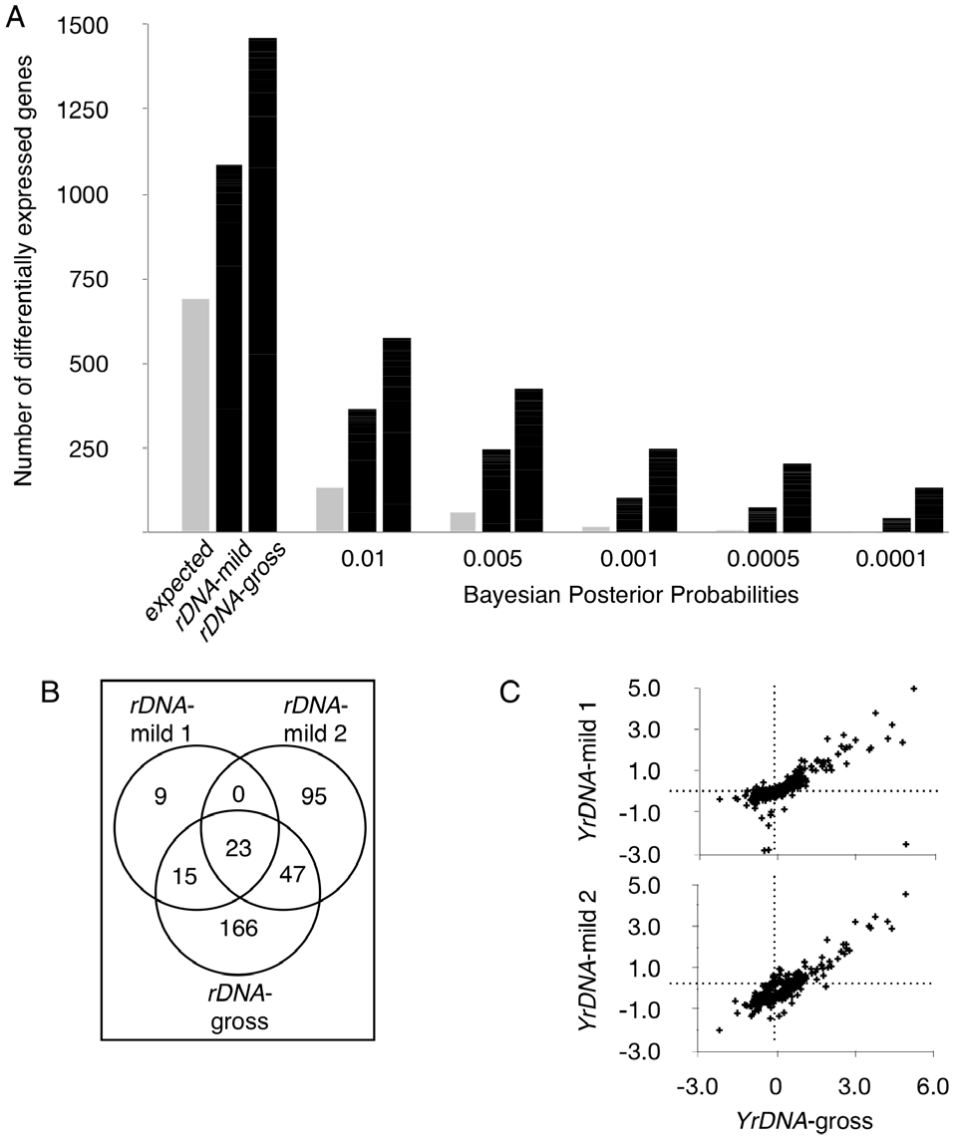
Induced deletions in the rDNA locus result in the differential expression of hundreds of genes. (A) Number of differentially expressed genes for *Y* chromosomes bearing deletions within the ribosomal DNA (*rDNA*). Data are given at *P*<0.05 (first set of data) and other indicated Bayesian Posterior Probabilities. Expected values are calculated from permuted datasets and shown in light gray. *rDNA*-mild are average numbers for two chromosomes with 87% and 85% wild-type copy number of *rDNA*, and *rDNA*-gross is a chromosome with 46% of wild-type *rDNA* (Figure 6). (B) Venn diagram showing number of differentially expressed genes in each *rDNA* deletion line relative to the wild-type chromosome (at *P*<0.001, FDR<0.05). (C) Correlation between the magnitude of change in gene expression (log-fold-changes) for *YrDNA-*gross (abscissa) and either *YrDNA-*mild-1 (ordinate – Top panel) or *YrDNA-*mild-2 (ordinate – Bottom panel). ρ = 0.84 and 0.78, respectively. Fold-changes are for contrasts between each *rDNA* deletion line and the wild-type chromosome.

Four *Y* chromosomes that differ in the copy number of *rDNA* repeats were introgressed into an isogenic background of autosomes and *X* chromosome (Figure S1); genome-wide expression was interrogated with microarrays (Figure S2). We compared adult male flies bearing a wild-type *Y* chromosome to three strains carrying mutant derivative *Y* chromosomes with reduced *rDNA* repeats – two mildly (*YrDNA*-mild, with *rDNA* copy numbers approximately 87% and 85% of wild-type) and one grossly (*YrDNA*-gross, 46% of wild type) reduced array. We observed substantial gene expression variation among these strains as compared to the random expectation across a range of *P*-values (Figure 1A). We expected that laboratory induced deletions in the *rDNA* array would result in gene expression modulation that would not only be replicated in each independently generated mutant, but would also be more pronounced in mutants lacking a larger proportion of the original locus. Our data are in agreement with both these expectations, and further suggest that *rDNA* copy number may be the major source of variation between these chromosomes. Accordingly, the numbers of differentially expressed genes were positively correlated with the *rDNA* deletion size. We observed that the *Y* chromosome with the lowest *rDNA* copy number (*YrDNA*-gross) induced the highest number of expression changes, whereas the two *Y* chromosomes with mildly-deleted arrays resulted in smaller numbers of differentially expressed genes. This finding is consistent with evidence that chromosomes with fewer *rDNA* copies have a stronger effect on position effect variegation [10]. Furthermore, the genes identified as differentially expressed in the mild deletions were a subset of those induced in the strain with grossly deleted *rDNA* (Figure 1B). Accordingly, 42–71% (*P*<0.001, FDR <0.05) of the genes identified with chromosomes containing mild *rDNA* deletions were also identified by the *Y* chromosome with the smallest *rDNA* copy number. In support of the reproducibility of the gene expression modulation that was observed in PEV across the lines [10], 24% of differentially expressed genes were shared by at least two chromosomes with reduced *rDNA* arrays, whereas fewer than 0.2% were expected to be shared by chance in randomly permuted datasets (P<0.001). In addition, the direction and magnitudes of changes in expression were significantly correlated (ρ = 0.78–0.84, *P*<10E−16) between *Y* chromosomes harboring *rDNA* deletions (Figure 1C), suggesting a similar responsiveness of the affected genes regardless the size of the *rDNA* deletion. These data support our contention that a common lesion – namely *rDNA* copy number variation – underlies this phenotype, and points to the relevance of *rDNA* copy number variation to modulation of genome-wide gene expression.

Natural polymorphisms and induced deletions of the *rDNA* show no overt dominant phenotype [3], [17]. Even *X0* males, which other than being sterile due to loss of *Y*-linked fertility genes, appear morphologically normal despite having no *Y*-linked *rDNA* [9]. Therefore, we expected that induced *rDNA* deletion would have impacts on individual gene expression that were generally small. Indeed, we found that 85% of genes whose expression differed significantly from wild-type had changes in expression level of no more than 50% (Figure S3A). It was unexpected and unlikely that the number of genes showing only a 10% change in relative expression would be less than those showing a 20% change. Instead this result is likely an underestimation of genes exhibiting small changes caused by limitation of statistical power to detect them. We projected a linear regression of the number of genes whose differential expression was altered by 10–20%, 20–30%, 30–40%, and 40–50% in an attempt to correct the first decile (Figure S3B–S3D). From that extrapolation, we estimate that as many as 1200–1700 differentially expressed genes at P<0.05 and 360–435 at P<0.01 might escape statistical detection despite our high level of replication (Figure S2). Since we analyzed 8073 unique gene IDs from our microarrays, these estimates suggest that as much as 40% of the genome might be subtly affected by partial *rDNA* deletion.

**Figure 2:**
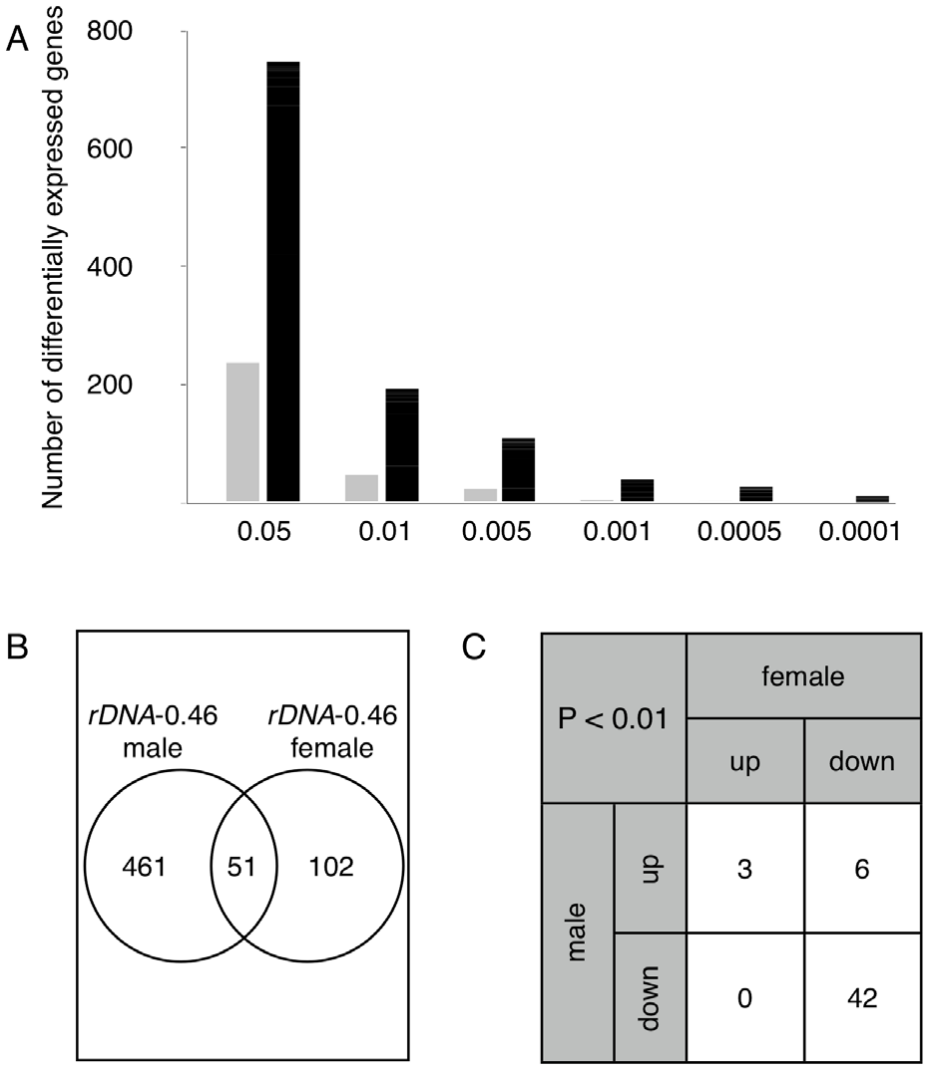
Differentially expressed genes are shared in males and females. (A) Number of differentially expressed genes in *XX*/*YrDNA*-gross females (relative to the wild-type *Y* chromosome in *XX/Y* females). Data are presented as in Figure 1A. (B) Venn diagram showing number of differentially expressed genes that are unique or common to *X*/*YrDNA*-gross males and *XX/YrDNA*-gross females (at *P*<0.001). (C) Breakdown of overlapping genes from (B), separately categorizing genes whose expression was increased (up) or decreased (down) relative to the wild-type *Y* chromosome in the same genetic background.

To determine the generality of *rDNA* induced expression changes, we investigated the differential expression between the *rDNA*-deleted *YrDNA-*gross and its ancestral wild-type *Y* chromosome in females with *XXY* karyotype. We observed hundreds of gene expression differences between *XXYrDNA-*gross and isogenic *XXY* females bearing the wild-type *Y* chromosome. The number of differentially expressed genes was fewer in *XXY* females than in males (Figure 2A), possibly due to the smaller fractional *rDNA* size difference in the female karyotype which contains two wild-type *X*-linked arrays. Nevertheless, we observed a significant enrichment of differentially expressed genes commonly shared between males and females harboring the same *Y* chromosome; at *P*<0.01, 51 genes were shared between the sexes whereas only a single gene was expected to be shared by chance alone. As before, expected numbers were calculated from randomly permuted datasets (Figure 2B, *cf.* Figure S4A). While the number of affected genes differed between the sexes, we observed a significant association between down-regulated genes (Figure 2C, Figure S5), and a significant correlation in fold-changes between sexes (ρ = 0.45, *P*<10E–16). A similar trend of up- and down-regulation and commonly shared genes remained across a range of *P*-values used for ascertaining differential expression (Figure S4B, S4C). Together, our data show that *rDNA* copy number variation commonly affects male and female transcription and identifies a similar set of “*rDNA*-sensitive” genes. These data suggest that the response of a gene to *rDNA* deletion is an attribute of the gene structure and/or its regulation, rather than a sex-dependent effect.

**Figure 3:**
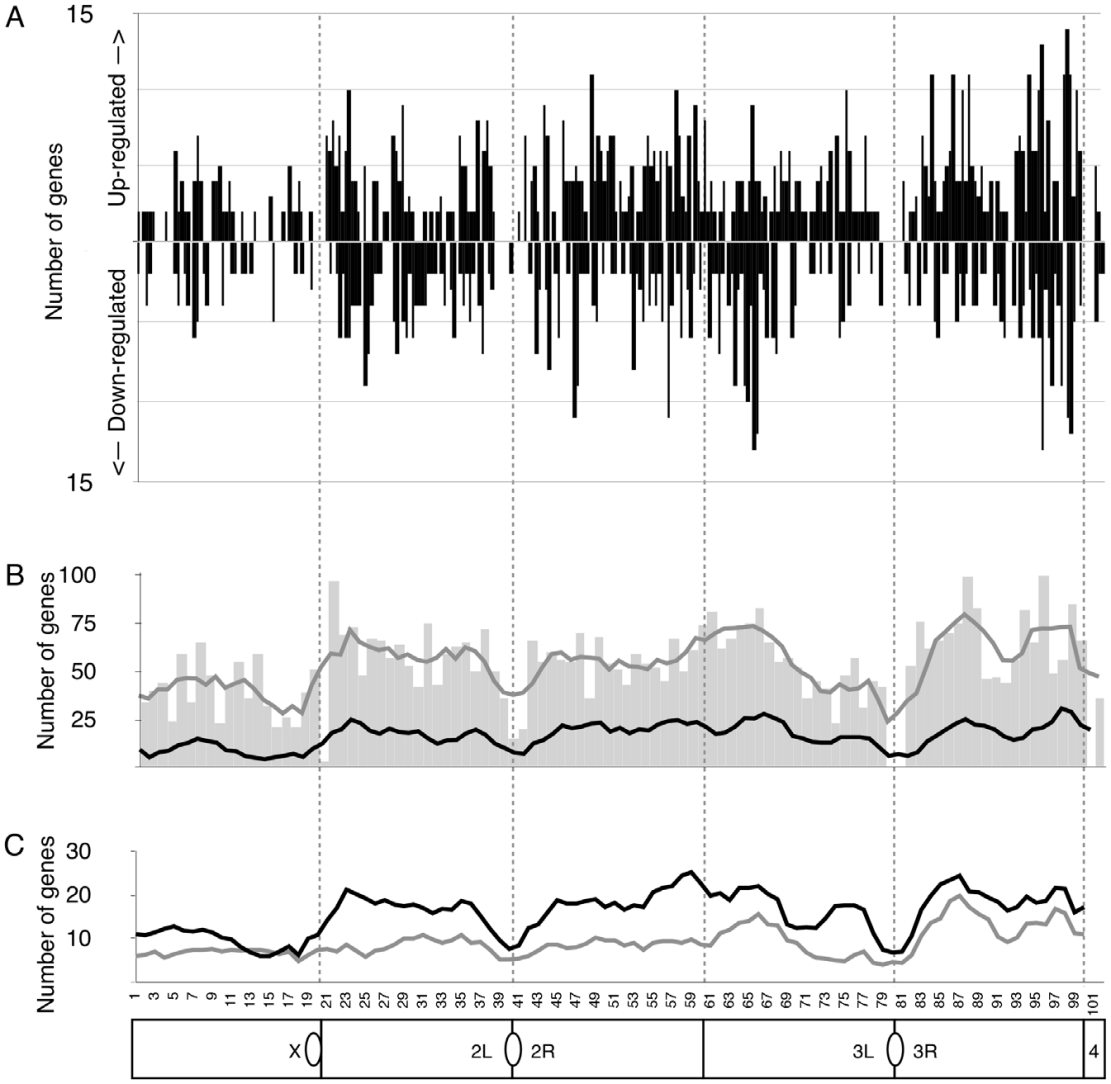
*rDNA*–responsive genes are found throughout the genome. (A) Number of differentially expressed genes either up-regulated or down-regulated as a function of cytological location. Each cytological division shows grouped data for all three *Y* chromosomes (*YrDNA*-mild-1, *YrDNA*-mild-2, *YrDNA*-gross) relative to the wild-type *Y* chromosome (at *P*<0.01). (B) Distribution of microarray spots yielding high quality data (gray bars) with scanning 5-division average (gray line). Overlaid scanning 5-division average (black line) of the number of differentially expressed genes. For each window we show the number of differentially expressed genes grouped for all three chromosomes (*YrDNA*-mild-1, *YrDNA*-mild-2, and *YrDNA*-gross) relative to the wild-type *Y* chromosome (*P*<0.01). (C) Scanning 5-division average of number of differentially expressed genes only from males (black) and females (gray) bearing *YrDNA*-gross (at *P*<0.01). Cytological divisions are aligned across entire figure (dotted vertical lines). Stylized chromosome map represents euchromatic regions of the genome and location of centromeres and centric heterochromatin (ovals).

One mechanism for the wide-ranging effects of *rDNA* copy number on gene expression might arise if deletions of the *rDNA* compromised the spread of heterochromatin structure to euchromatin from centric heterochromatic regions [18], [19]. Indeed, this simple model is suggested by the observation that three heterochromatin-induced variegating alleles were affected by *rDNA* deletion [10]. However, studies have established that the spreading of heterochromatin-associated factors is neither long-ranged, processive, or simple [20], [21]. Nonetheless, to address the issue we tested two strong predictions of a simple heterochromatin spreading model: that (i) genes residing in the proximity of heterochromatin will be more strongly affected by changes in *rDNA* copy number, and that (ii) the majority of gene expression changes will be seen as increase in expression as repressive heterochromatin is reduced. We therefore tested for an enrichment of differentially expressed genes according to their cytological location by plotting the number of differentially expressed genes from all three chromosomes (Figure 3A black bars, Figure 3B black line). We compared the number of differentially expressed genes to the number of analyzed genes from the microarray (Figure 3B gray bars and line), which corrected for uneven gene density across the genome and for any bias in the representation of the genome on our microarrays.

We found that while the number of differentially expressed genes appears to drop near the cytological bands juxtaposed to centric heterochromatic blocks (bands 20, 40/41, and 80/81), this is indistinguishable from our expectation based on lower representation of genes from these regions. Moreover, we could discern no general trend for genes near the telomeres (bands 1, 21, and 60/61). Furthermore, while *X*-linked loci were less affected than were chromosome *2*- and *3*-linked genes (43–47% the frequency), these trends were not statistically different from that expected given the distribution of represented genes (Figure S6A). Next, we analyzed the genomic region at the transition between the *X*-linked 20 megabases of centric heterochromatin and adjacent euchromatin. None of the first 20 genes were differentially expressed. The same held true for the telomere of the long arm of the *X* chromosome. Finally, we assessed the density of genes responding to *rDNA* copy number along all the chromosome arms in a sliding 2 megabase window at 1 megabase increments. Significance was determined empirically by analyzing 1000 sets of randomly sampled genes taken from the entire genome. Each set of random data had a sample size equal to the number of differentially expressed genes. No significant overrepresentation of differentially expressed genes was detected in any increment. Thus, our analysis indicated that the genes affected by *rDNA* deletion are randomly distributed in the genome, and are neither influenced by proximity to heterochromatin nor chromosome linkage. There was also no preponderant increase or decrease of differential expression. Corroborating our conclusion that *rDNA* deletion affects the same set of genes in males and females, we saw that the distribution of affected genes in males and females was coincident (Figure 3C, Figure S6B). Taken together these results argue against a simple heterochromatin spreading model but instead suggest that loci with sensitivity to *rDNA* copy number variation are scattered through the genome.

An intriguing possibility is that polymorphisms of the *rDNA* copy number in naturally occurring *Y* chromosomes could account in part for the differential genome-wide modulation of gene expression exerted by these chromosomes. One indication that such *rDNA*-driven *Y*-linked variation is relevant came from categorizing the differential expressed genes by Gene Ontogeny (GO) category. These analyses pointed to five categories that overlapped with those discovered by comparing differential gene expression due to natural *Y* chromosome polymorphisms (Table 1) [11]. Importantly, energy metabolism and *rDNA* activity are closely associated [22], [23]. Our data suggest that *rDNA* copy number itself might play a role regulating energy metabolism through modulation of gene expression of genes responsible for energy management or membrane metabolism. Accordingly, we observed that in both males and females “*rDNA* sensitive” genes are highly enriched for proteins localized to the mitochondrial membrane (46 genes in males, *P*<2.1E–10; 38 genes in females *P*<7.2E–12) and involved in electron transport (44 genes in males, *P*<2.3E–05; 38 genes in females, *P*<1.4E–09). These enrichments are significant in both males and females, but collectively account for 16% and 9% respectively of the differentially expressed genes (at *P*<0.05). Although energy and lipid metabolism may be influenced by *rDNA* copy number, they are not the sole targets.

**Table 1:** *rDNA* copy number affects genes related to energy metabolism.

	*Category*	*Description*	*N*	*P-value*
male	GO: 0031966	Mitochondrial membrane	46	2.1E−10
	GO: 0006118	Electron transport	44	2.3E−05
	GO: 0016298	Lipase activity	16	1.1E−03
	GO: 0006629	Lipid metabolic process	53	1.4E−03
	GO: 0006631	Fatty acid metabolism	9	1.8E−02
female	GO: 0031966	Mitochondrial membrane	38	7.2E−12
	GO: 0006118	Electron transport	38	1.4E−09

Gene Ontogeny (GO) categories whose genes are disproportionally represented among those with expression significantly altered in males (top) and females (bottom). *P*-values are corrected for multiple testing using the modified Bonferroni correction of the statistical software package GeneMerge.

**Figure 4:**
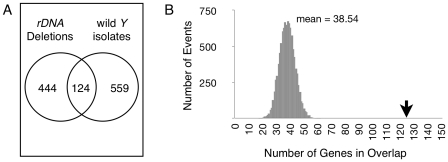
Differentially expressed genes are shared between chromosomes with induced *rDNA* deletions and naturally occurring *Y* chromosomes. (A) Venn diagram showing number of differentially expressed genes unique to *Y* chromosomes with induced *rDNA* copy number changes (“*rDNA* Deletions”) or natural *Y* chromosomes (“wild *Y* isolates”), and overlap of genes common to both groups (at *P*<0.005). (B) Event histogram showing that 10,000 randomly-generated datasets produces an average of 38.54 genes shared between *rDNA* Deletions and wild *Y* isolates. Arrow shows the observed value of 124 (from (A)).

To address the relevance of *rDNA* copy number in natural populations, we integrated data from gene expression variation due to natural *Y*-linked polymorphisms and gene expression variation due to deletions in the *rDNA* array. We found an unexpected level of overlap in the identity of differentially expressed genes when compared to the level expected by chance alone (Figure 4A, Figure S7). We found 124 genes with evidence for differential expression (*P*<0.005) in comparisons between chromosomes with induced changes in *rDNA* copy number and in comparisons between naturally-occurring chromosomes. We assessed statistical significance of this overlap in two ways. First, the same procedure for identifying differential expression was applied to datasets in which the expression data were randomly permuted. These analyses showed that only five genes were identified as differentially expressed in both permuted datasets. Second, we generated random samples of size 568 (the observed number of differentially expressed genes shared between the induced *rDNA* chromosomes) and 683 (the observed number of differentially expressed genes shared between the natural chromosomes), and counted the number of genes that appeared in the overlap of both sets in each of 10,000 replicated of this experiment. The analysis indicates that the observed overlap of 124 genes is significantly enriched compared to random datasets (*P*<0.0001, Figure 4B). This enrichment shows that a gene affected by YRV is predictive of being affected by *rDNA* copy number.

**Figure 5:**
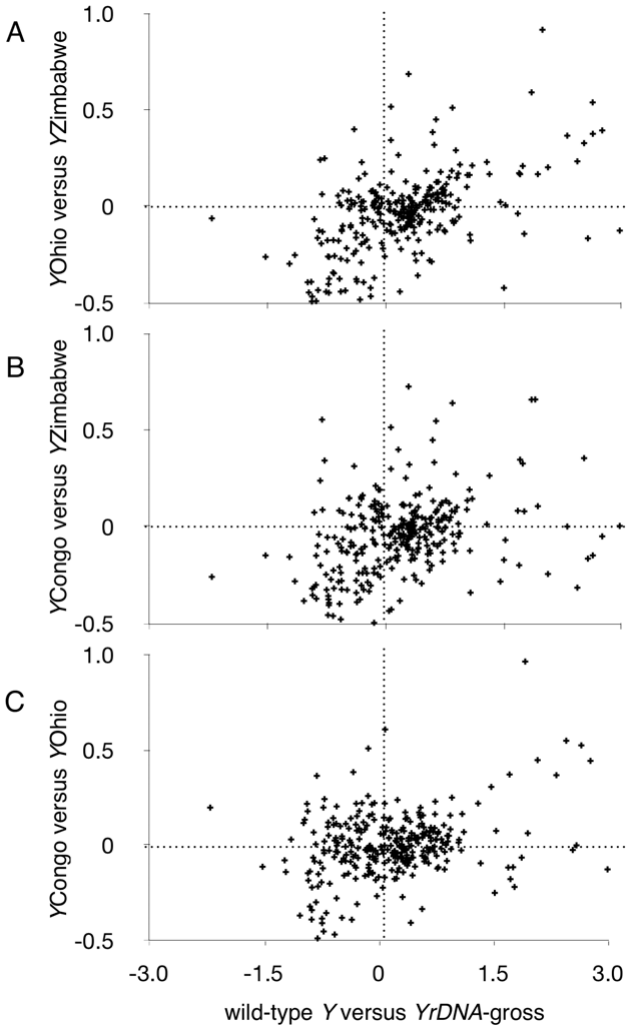
Correlations between absolute fold changes in *rDNA* responsive genes identified by induced *rDNA* deletions and absolute fold changes arising from natural *Y* chromosome polymorphisms. (A) Correlation of absolute log-fold-changes comparing differentially expressed genes between *YrDNA-*gross and wild-type *Y* (abscissa) to those differentially expressed between *Y*Zimbabwe (*Y*Zimb.) and *Y*Ohio (ordinate); ρ = 0.55, *P*<10E−16. (B) Correlation of absolute log-fold-changes for *YrDNA-*gross versus wild-type *Y* compared to *Y*Congo versus *Y*Zimbabwe; ρ = 0.38, *P*<10E−12. (C) Correlation of absolute log-fold-changes for *YrDNA-*gross versus wild-type *Y* compared to *Y*Congo versus *Y*Ohio; ρ = 0.25, *P*<10E−6.

**Figure 6:**
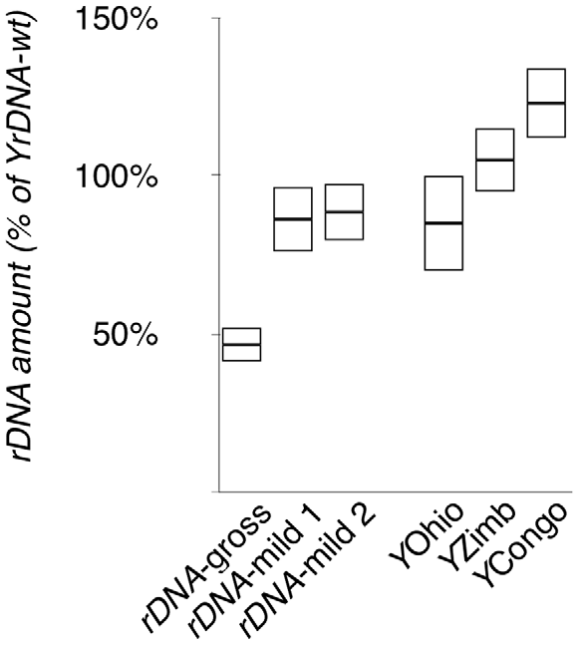
Quantification of *rDNA* copy number of the chromosomes in this study. Quantification of *rDNA* copy number determined by Real Time Polymerase Chain Reaction, presented as percentage of a common wild-type *Y* chromosome (the progenitor of *YrDNA*-mild-1, *YrDNA*-mild-2, and *YrDNA*-gross). Plots show average ± 1 S.D.

Finally, we analyzed the set of genes whose expression is affected by *rDNA* deletion, and found that absolute fold changes due to these deletions is significantly correlated with expression variation arising due to naturally-occurring *Y* chromosome polymorphisms (ρ = 0.25–0.55, *P*<1.0E–12, Figure 5). These correlation coefficients indicate that approximately 5–30% of gene expression variation detected on natural *Y* chromosomes might be due to polymorphisms in the *rDNA* loci. Using real-time PCR we confirmed that the natural *Y* chromosomes possessed polymorphisms in *rDNA* copy number, the range of which included the two mild *rDNA* deletions used in this study (Figure 6). Moreover, the effect of naturally occurring *Y* chromosomes from *Y*Ohio and *Y*Zimbabwe on heterochromatin-induced position effect variegation were consistent with effects seen for induced deletions: lower *rDNA* copy number correlated with increased suppression of variegation [10], [12].

## Discussion

Our work identified the consequences of induced *rDNA* deletions to the expression of hundreds to thousands of genes scattered through the genome of both males and females. The data are reproducible between independently-generated chromosomes derived from a common ancestor, with lines containing smaller deletions showing smaller effects on gene expression. Although we cannot exclude the possibility that some undetected *Y*-linked differences other than *rDNA* copy number might confound some of the effects we have seen, we interpret the changes in gene expression to be, at least in part, a consequence of induced deletions in the *rDNA* locus. Previous studies lend credence to this interpretation [10], [14], [17]. Furthermore, the results herein reported are well in line with the expectation that the measured *rDNA* deletions partly underlie the expression phenotypes we report. Accordingly, we observed that the *Y* chromosome with the lowest *rDNA* copy number (*YrDNA*-gross) induced the highest number of expression changes, whereas the two *Y* chromosomes with mildly-deleted arrays resulted in smaller numbers of differentially expressed genes. This finding is consistent with evidence that chromosomes with fewer *rDNA* copies have a stronger effect on position effect variegation [10]. Furthermore, the genes identified as differentially expressed in the mild deletions were a subset of those induced in the strain with grossly deleted *rDNA*. Furthermore, we showed that such *rDNA*-responsive genes are not physically clustered or near heterochromatin; instead, affected genes are distributed across the genome and show functional enrichments for genes encoding proteins localized to the mitochondria and involved in electron transfer. This finding is reminiscent of previous observations for a close link between *rDNA* activity and energy metabolism. Furthermore, our analyses suggested that naturally occurring copy number polymorphism in the *rDNA* array might be an important and underappreciated source of regulatory variation in natural populations.

Epigenetic regulation of chromatin structure is necessary for the stability of the *rDNA* array and the nucleolus. Indeed, destabilization of *rDNA* copy number and nucleolar structure are markers of aging and cancer progression [24], [25] and our findings raise the possibility that *rDNA* alterations might underlie some of these pleiotropic phenotypes through its consequences on genome-wide gene expression. Mechanistically, the transcriptional activity of the *rDNA* locus is regulated through histone modifications and allows for nucleolus formation from even a single *rDNA* repeat unit [8], [26]–[28]. Furthermore, many chromatin components known to regulate *rDNA* activity and stability are dosage sensitive. For instance, the Sir2-family proteins are required for the silencing of genes inserted into the *rDNA* repeated array, *rDNA* stability, and telomeric silencing [29], [30]. In *Drosophila*, Sir2 has an essential role in both euchromatic gene repression and heterochromatic silencing [29]. Hence, it is conceivable that *rDNA* expression and *rDNA* copy number are related, and perturbations to either might yield heritable alteration in genome structure with consequences on gene expression of unlinked genes. Accordingly, we propose that short and long *rDNA* arrays may result in the differential recruitment of nuclear proteins for rRNA expression/processing and nucleolar structure. In such a model, variation of protein dosages due to either the release of proteins required for silencing of non-expressed *rDNA* repeats (which might be more abundant in the larger array) or due to the recruitment of proteins required for the higher expression of a smaller number of expressed *rDNA* repeats (as might be the case in the shorter *rDNA* arrays) might be expected. Indeed, Michel and colleagues showed that spontaneous *rDNA* deletions in yeast caused decreased cellular abundance of Sir2 mRNA and protein [31]. Hence, the widespread effects of *rDNA* copy number on genome-wide expression might arise from imbalances in the nuclear protein pool, which includes chromatin and transcription factors, that might result from the requirements for silencing and expression of variable numbers of *rDNA* repeats or structural elements within nucleoli. Alternatively, Paredes and Maggert showed alterations in nucleolus stability as a consequence of reduced *rDNA* copy number [10]. The dynamic composition of the nucleolus [32] may differ in nucleoli coalesced around long or short *rDNA* arrays.

Paredes and Maggert showed that *rDNA* copy number altered the balance of euchromatin and heterochromatin in *Drosophila* models of position effect variegation [10]. Those observations suggested a global effect of *rDNA* copy number on chromatin structure. Significantly, multiple studies show a complex regulation of heterochromatin spreading at natural euchromatin-heterochromatin transition zones and chromosome rearrangement breakpoint junctions. Yasuhara and Wakimoto showed that increased levels of HP1 association at variegating euchromatic-heterochromatic junctions is not homogenous across the chromosome [20], and both Talbert and Henikoff, and Ahmad and Henikoff showed that heterochromatin spreading does not translate into uniform responses for closely-linked genes [33], [34]. Most recently, Vogel and colleagues showed that while a variegating *white* ^+^ gene was responsive to changes in chromatin environment, the majority of linked genes remained unresponsive to the spreading of HP1 from nearby heterochromatin [21]. Hence, it was not surprising that genes responding to *rDNA* copy number were distributed across the genome. More important, this observation does not contradict the view that *rDNA* copy number impacts global chromatin structure. To the contrary, recent studies suggested that heterochromtain-induced PEV [12], [13] and euchromatic gene expression might be related [11], and *rDNA* deletions affect heterochromatin-induced PEV [10]: the data we present here supports the unity of these observations by showing that deletions within the *rDNA* affect euchromatic gene expression, uncover a significantly overlapping subset of genes, and account for a fraction of naturally occurring YRV.

Although *rDNA* copy number polymorphism may only account for a small fraction of YRV, our data indicate a relevant contribution with significant enrichments in the number of differentially expressed genes that are shared by both *rDNA* deletions lines and naturally occurring *Y* chromosomes. This is unexpected because *rDNA* copy number is typically thought to be without genetic consequence. The remainder of YRV may map to other heterochromatin of the *Y* chromosome, either simple repeats or transposable elements. Similarly, the remainder of *rDNA*-linked variation may be ameliorated or epistatically masked by compensatory polymorphisms elsewhere in the heterochromatin. Finally, our results raise the issue of how widespread these effects may be in other chromosomes and organisms containing highly variable *rDNA* loci. Indeed, the large and varied number of *rDNA* copies is a common characteristic of eukaryotic genomes [2]. The human diploid karyotype has ten *35S* and two *5S* arrays, each highly variable with regard to size and activity [35]. In plants such as *Arabidopsis*, the *5S rDNA* arrays contain heritable regulatory chromatin structure [36], and in flax, heritable induced phenotypic variation is mapped to *rDNA* copy number [37], [38]. Although *rDNA* copy number in *Drosophila* may vary over an order of magnitude, the upper limits remain consistent across many studies [1]. What limits the expansion of *rDNA* is yet to be established, but our results suggest that superfluous *rDNA* repeats – inactive or active – have genetic consequence to gene expression, which might impose a heretofore underappreciated pressure against excessive expansion or contraction. We envision this genetic consequence to be heritable as a result of copy number polymorphism, suggesting that *rDNA* copy number is an important consideration in understanding the evolution of a genome. Indeed, the *rDNA* array has a uniquely dynamic biology of copy number loss and gain that occurs meiotically and somatically through development [5], [39]–[41]. Hence, the ability of the *rDNA* to change in copy number and activity might provide a novel mechanism for adaptation to environmental changes by maintaining a euchromatin/heterochromatin balance that is most conducive for fitness.

## Materials and Methods

### Drosophila stocks and crosses

*Drosophila melanogaster* Y-chromosomes containing targeted deletions in the *rDNA* locus were generated as previously described [17]. These chromosomes were derived from a common single male ancestor in the laboratory that was subjected to targeted deletions within the *rDNA* locus, and thus created an allelic series of *Y* chromosomes virtually identical with the exception of variation in *rDNA* copy number. These *Y* chromosomes were introgressed into an isogenic (*X* chromosome, autosomes, and mitochondrial genome) laboratory stock [11] by crossing *XY* males to females homozygous for the markers *yellow, brown, ebony*, *cubitus interruptus*, and *eyeless* (*y/y; bw/bw; e/e; ci ey/ci ey*) for two generations (Figure S1). This isogenic stock is expected to contain very little genetic variation, and upon receipt was subjected to no fewer than eight additional generations of brother-sister mating to reinforce homozygosity of the genetic background. Four *Y* chromosomes were analyzed: The original *Y* chromosome that contains a wild type *rDNA* array (100%), two derived chromosomes with mild deletions 87% (*YrDNA*-0.87) and 85% (*YrDNA*-0.85) of wild-type, and one grossly reduced derived chromosome that contains 46% (*YrDNA*-0.46) of wild-type. Flies were grown under 24 h light at constant temperature (25°C) and humidity (80%). *XXY* female flies were obtained by crossing males from the isogenic *Y* chromosome substitution lines described above to females from a laboratory stock containing a compound (attached) *X* chromosome, *C(1)M4, y*.

### *rDNA* copy number analyses

*rDNA* copy number in *Y*-chromosome substitution strains containing the *rDNA* deletions were checked with a random subset of flies used for gene expression analysis. *rDNA* copy number was measured as described in Paredes and Maggert [17]. For each stock whose *Y*-linked *rDNA* array was measured males were crossed with *C*(*1*)*DX*, *y* ^1^ *f* ^1^ *bb* ^0^, which lacks all *X*-linked *rDNA*. The resulting *C*(*1*)*DX*/*Y* females contain only *Y*-linked *rDNA*, whose copy number was determined with quantitative Real-time PCR using conserved *rDNA* primers that amplified *18S* subunit rRNA gene sequence. For Real-time PCR analyses, five biological replicates consisting of three experimental replicates from individual flies were sampled for each genotype. Real-time PCR analyses were carried out with the Power Sybr Green Master Mix (Applied Biosystems, Foster City, California), and amplification profiles were obtained with an ABI Step-One Real-time PCR (Applied Biosystems, Foster City, California). Reactions were quality-confirmed for the absence of dimers or nonspecific amplification by analyzing melting curve kinetics and electrophoretic analysis of products.

### Gene expression analyses

Microarrays were approximately 18,000-feature cDNA arrays spotted with *Drosophila melanogaster* cDNA PCR products. For RNA extraction, newly emerged male flies were collected and aged for three days at 25°C, after which they were flash frozen in liquid nitrogen and stored at −80°C. When females were analyzed, they were collected within 7 hours of eclosion to assure they were unmated prior to aging under the same conditions as were males. Total RNA was extracted from whole flies using TRIZOL (Gibco-BRL, Life Technologies, Gaithersburg, Maryland). cDNA synthesis, labeling with fluorescent dyes (Cy3 and Cy5) and hybridization reactions were carried out using 3DNA protocols and reagents (Genisphere Inc., Hatfield, Pennsylvania). Slides were scanned using AXON 4000B scanner (Axon Instruments, Foster City, California) and the GenePix Pro 6.0 software. Stringent quality-control criteria were used to ensure reliability of foreground intensity reads for both Cy3 and Cy5 channels. Foreground fluorescence of dye intensities was normalized by the Loess method in the library Limma [42], [43] of the software R. Significance of variation in gene expression due to *Y* chromosome origin was assessed with linear models and empirical Bayes moderated F statistics in Limma [42], [43]. *P* values were adjusted for multiple testing by using the method of Benjamini and Hochberg to control the false discovery rate [44]. Test results were considered to be significant if the adjusted *P* values were less than 0.05, nominally controlling the expected false discovery rate to no more than 5%. Differential expression was also assessed using the Bayesian Analysis of Gene Expression Levels (BAGEL) model [45]. Results were robust to the choice of linear models in Limma or BAGEL. False discovery rates were estimated based on the variation observed when randomized versions of the original dataset were analyzed. Similarly, expected values for the overlap between independent datasets were estimated by applying the same statistical procedure (Limma or BAGEL) to permuted versions of the datasets. Significance of the overlap was further assessed by generating samples of sizes that matched the number of differentially expressed genes in separate datasets (induced *rDNA* deletions and natural *Y* chromosomes) and determining the number of genes found in both random samples; the mean number was 38.54, and the maximum was 62 genes in 10,000 trials. Enrichment in gene ontology categories was assessed using a modified Bonferroni correction with GeneMerge [46]. Microarray gene expression data can be obtained at the GEO database (GSE27695).

## Supporting Information

Figure S1 (A) Crossing scheme to introgress *Y* chromosomes to a common and isogenic genetic background. *y* (*yellow*), *bw* (*brown*), *e* (*ebony*), *ci* (*cubitus interruptus*), *ey* (*eyeless*) were used as recessive genetic markers. (B) Crossing scheme to generate *XX*/*Y* aneuploid females. Circle represents common centromere linking compound-*X* chromosome arms.
(0.30 MB TIF)

Figure S2 (A) Array design for comparing males. *rDNA* sizes were determined at the onset of the experiments. *rDNA*-mild-1 is approximately 87% the wild-type size, *rDNA*-mild-2 is 85% the wild-type size, and *rDNA*-gross is 46% the wild-type size (Figure 6). Lines are direct comparisons and indicate number of replicates. (B) Array design for comparing females.
(0.24 MB TIF)

Figure S3 (A) Number of differentially expressed genes (at *P*<0.01), broken down by decile “fold-changes” along the abscissa for the *YrDNA*-gross deleted chromosome, presented as absolute counts (solid lines and ordinal values) and cumulative percentage (dotted lines at 20% increments). (B) Data from (A) graphed as separate deciles to show quality of estimation. Log scale inset shows fold-changes 1.1 to 1.5. (C) Data from Figure 1A (*P*<0.05) graphed as separate deciles to show quality of estimation. Log scale inset shows fold-changes 1.1 to 1.5. (D) Number of differentially expressed genes (data from (B) and (C)) with estimated number of genes whose expression was modulated by less than 10% and missed due to limited statistical power (white). Data were generated from linear regression of subsequent four deciles. Projections are shown for *P*<0.05 (left) and *P*<0.01 (right).
(0.36 MB TIF)

Figure S4 (A) Expectation of overlap at *P*<0.01 based on chance alone. *cf.* Figure 2B. (B) Data from Figure 2A at *P*<0.005 versus values expected by chance alone. (B) Data from Figure 2A at *P*<0.005 versus values expected by chance alone. (C) For *P*<0.001. *cf.* Figure 2B, 2C.
(0.47 MB TIF)

Figure S5 Correlation of log-fold-changes comparing differentially expressed genes between *YrDNA*-gross and the wild-type *Y* in males (abscissa) to those differentially expressed between *YrDNA*-gross and the wild-type *Y* in females (ordinate); ρ = 0.45, *P*<0.0001.
(0.22 MB TIF)

Figure S6 (A) Ratio of number of differentially expressed genes to total number of genes on the microarray (data from Figure 3B). Solid horizontal line shows the average, dotted lines show two standard deviations. (B) Cumulative counts of differentially expressed genes between *YrDNA*-gross and wild-type *Y* (at *P*<0.01) in males (black), females (gray), and females multiplied by a correction (dotted gray) to more easily compare trends (data from Figure 3C).
(0.29 MB TIF)

Figure S7 (A) Observed number of shared differentially expressed genes across pairwise chromosome comparisons. *P*<0.05 data are shown above the diagonal, *P*<0.005 are shown below the diagonal, and total number (shared plus unique) of differentially expressed genes (*P*<0.05/*P*<0.005) are shown on the diagonal (bold, gray background). (B) Expected numbers of genes shared between induced *rDNA* deletion *Y* chromosomes and natural *Y* chromosomes, calculated from randomized datasets (N for individual pairwise comparisons are on diagonal) at *P*<0.05 (above diagonal) and *P*<0.005 (below diagonal).
(0.27 MB TIF)
