# Supplementary figures and images for: Ribosomal DNA Deletions Modulate Genome-Wide Gene Expression: “rDNA–Sensitive” Genes and Natural Variation
Source: PLoS Genet. 2011 Apr 21;7(4):e1001376. doi: 10.1371/journal.pgen.1001376 (PMC3080856; doi:10.1371/journal.pgen.1001376)

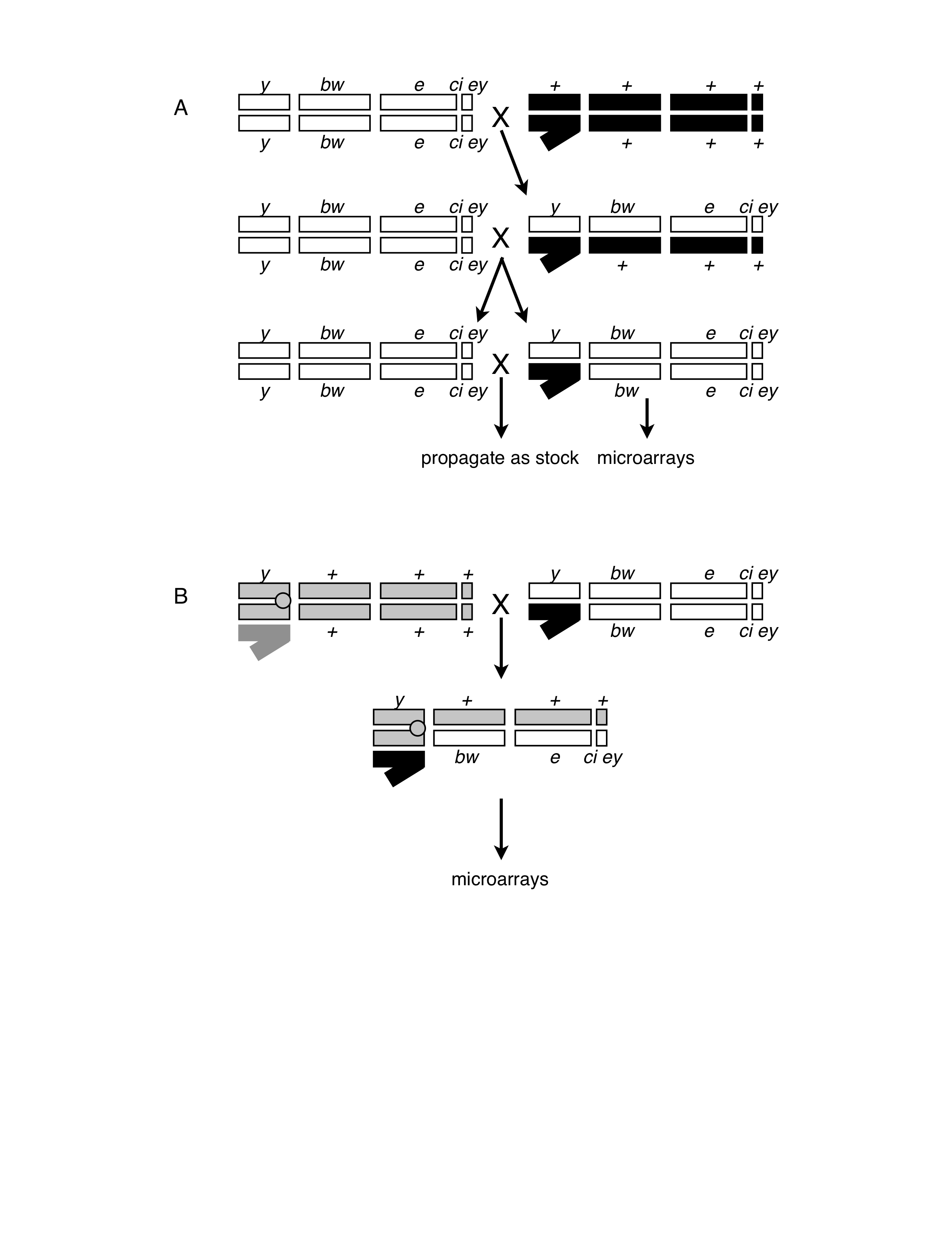

Supplement: Figure S1 — (A) Crossing scheme to introgress Y chromosomes to a common and isogenic genetic background. y (yellow), bw (brown), e (ebony), ci (cubitus interruptus), ey (eyeless) were used as recessive genetic markers. (B) Crossing scheme to generate XX/Y aneuploid females. Circle represents common centromere linking compound-X chromosome arms. (0.30 MB TIF) [file pgen.1001376.s001.tif]

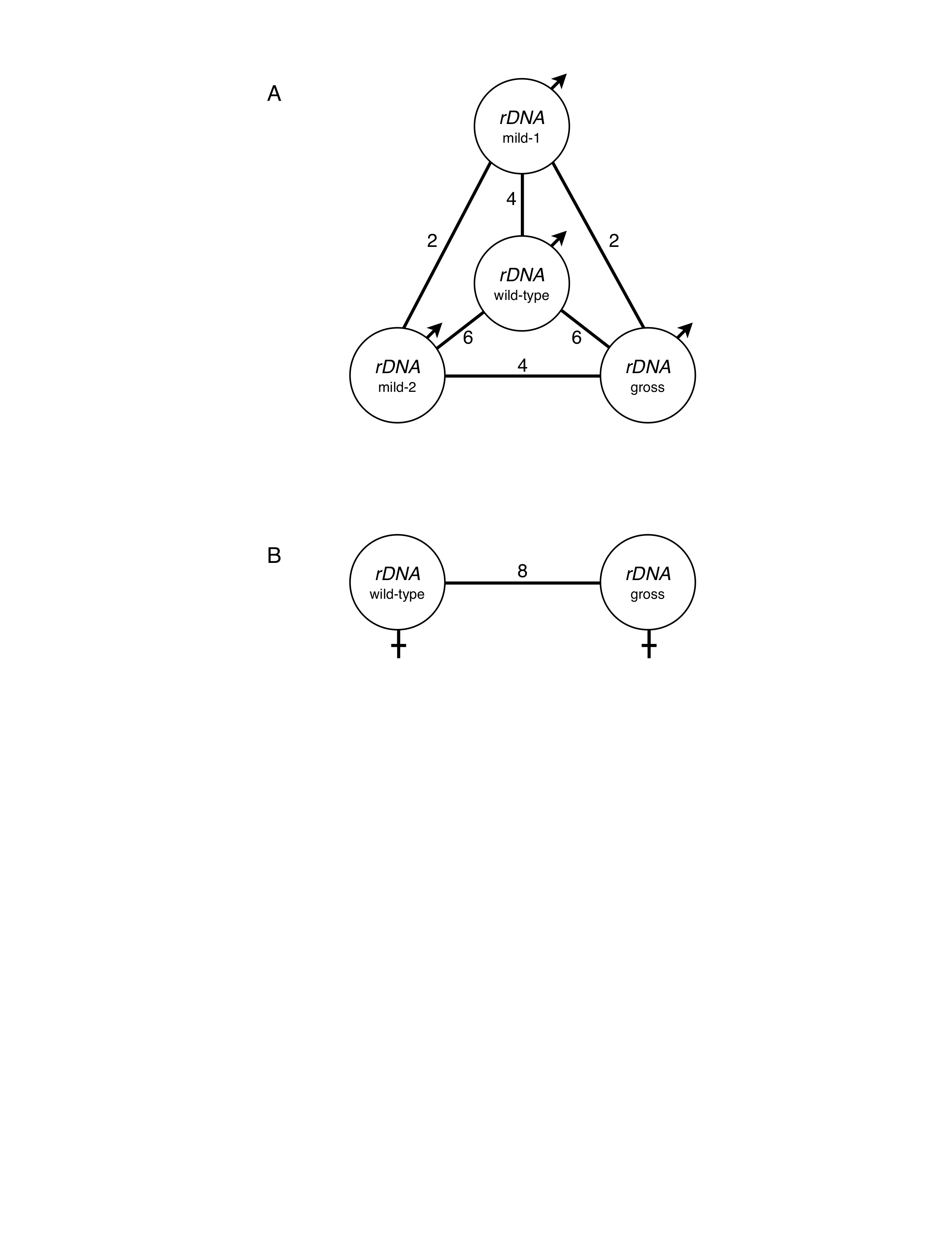

Supplement: Figure S2 — (A) Array design for comparing males. rDNA sizes were determined at the onset of the experiments. rDNA-mild-1 is approximately 87% the wild-type size, rDNA-mild-2 is 85% the wild-type size, and rDNA-gross is 46% the wild-type size (Figure 6). Lines are direct comparisons and indicate number of replicates. (B) Array design for comparing females. (0.24 MB TIF) [file pgen.1001376.s002.tif]

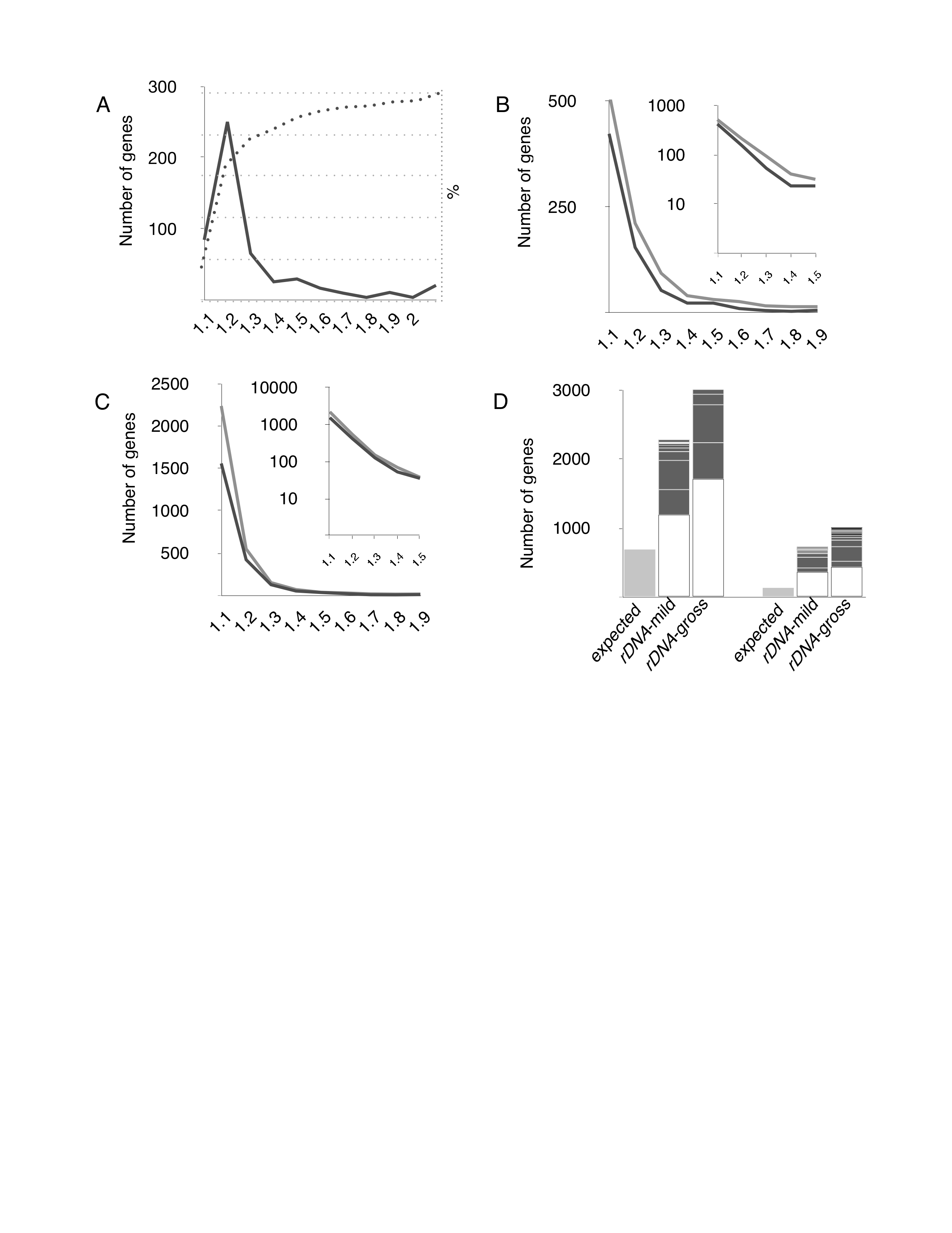

Supplement: Figure S3 — (A) Number of differentially expressed genes (at P<0.01), broken down by decile “fold-changes” along the abscissa for the YrDNA-gross deleted chromosome, presented as absolute counts (solid lines and ordinal values) and cumulative percentage (dotted lines at 20% increments). (B) Data from (A) graphed as separate deciles to show quality of estimation. Log scale inset shows fold-changes 1.1 to 1.5. (C) Data from Figure 1A (P<0.05) graphed as separate deciles to show quality of estimation. Log scale inset shows fold-changes 1.1 to 1.5. (D) Number of differentially expressed genes (data from (B) and (C)) with estimated number of genes whose expression was modulated by less than 10% and missed due to limited statistical power (white). Data were generated from linear regression of subsequent four deciles. Projections are shown for P<0.05 (left) and P<0.01 (right). (0.36 MB TIF) [file pgen.1001376.s003.tif]

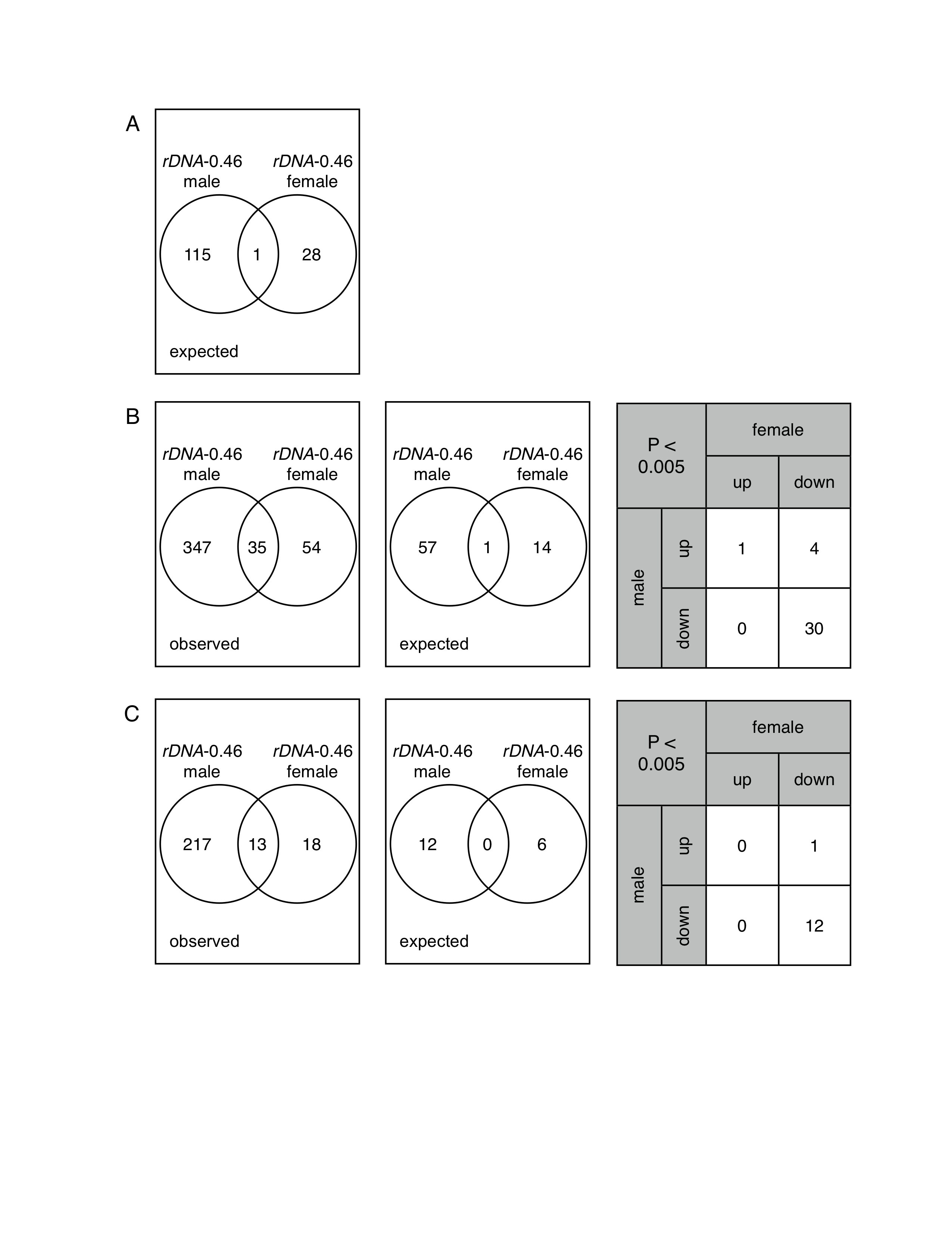

Supplement: Figure S4 — (A) Expectation of overlap at P<0.01 based on chance alone. cf. Figure 2B. (B) Data from Figure 2A at P<0.005 versus values expected by chance alone. (B) Data from Figure 2A at P<0.005 versus values expected by chance alone. (C) For P<0.001. cf. Figure 2B, 2C. (0.47 MB TIF) [file pgen.1001376.s004.tif]

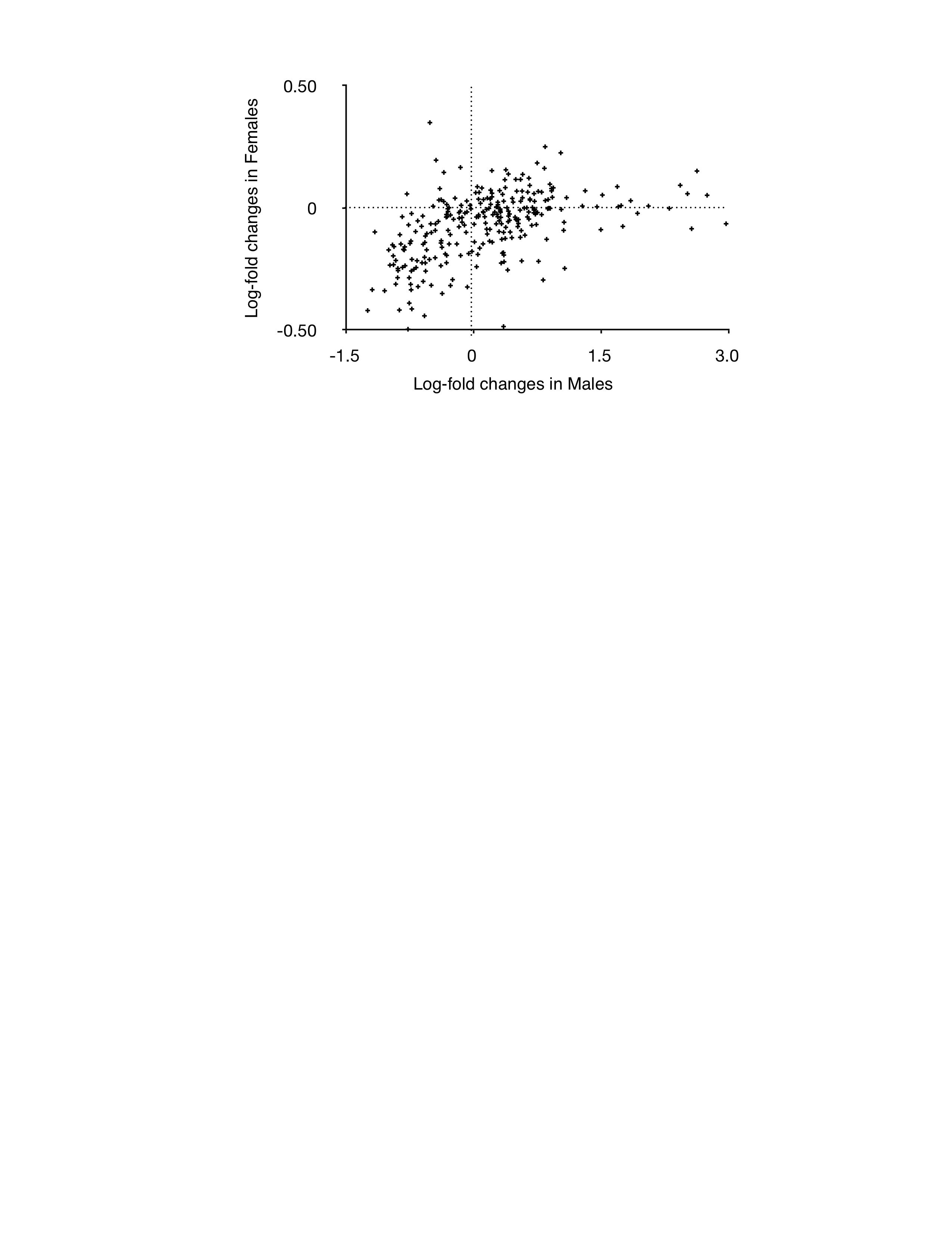

Supplement: Figure S5 — Correlation of log-fold-changes comparing differentially expressed genes between YrDNA-gross and the wild-type Y in males (abscissa) to those differentially expressed between YrDNA-gross and the wild-type Y in females (ordinate); ρ = 0.45, P<0.0001. (0.22 MB TIF) [file pgen.1001376.s005.tif]

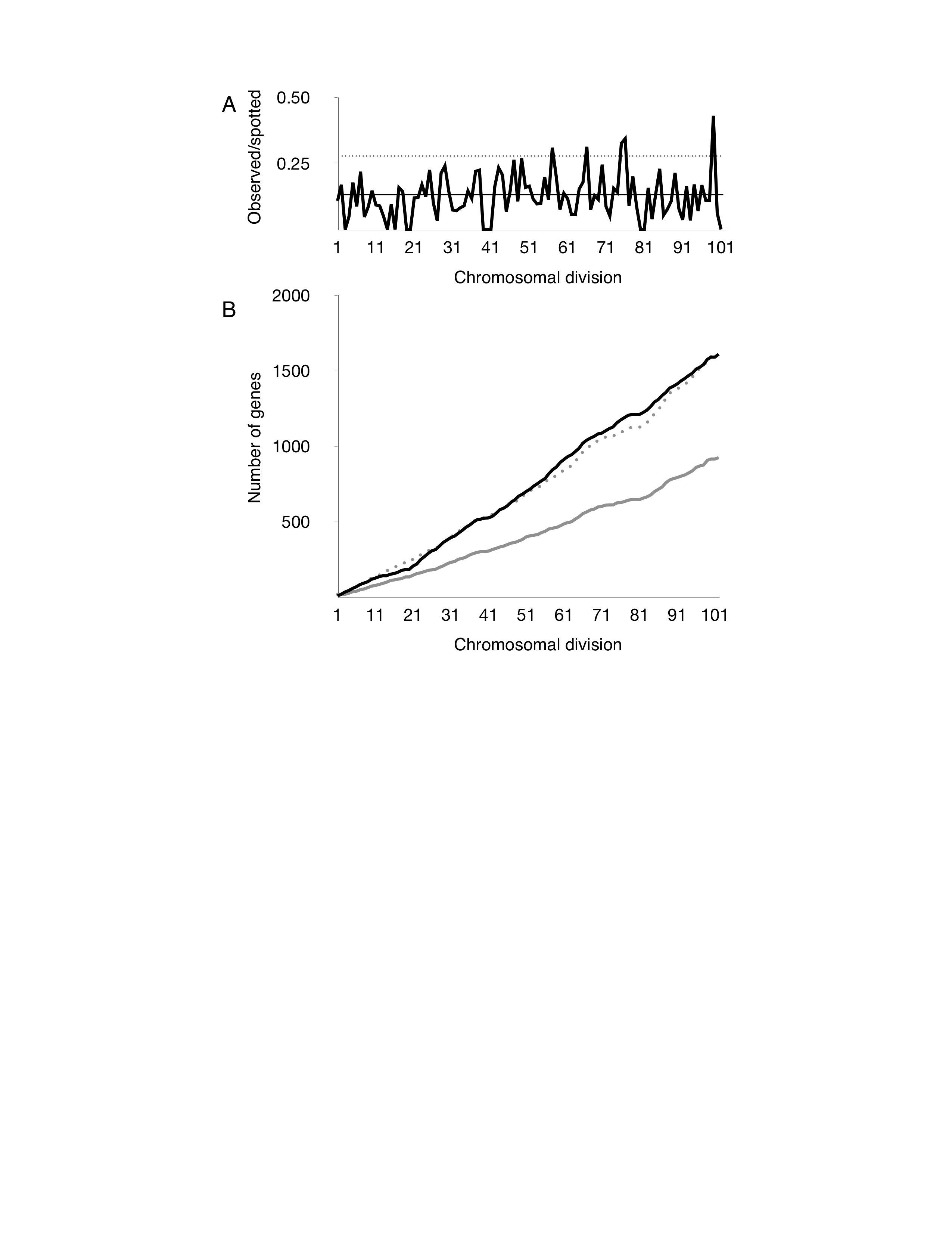

Supplement: Figure S6 — (A) Ratio of number of differentially expressed genes to total number of genes on the microarray (data from Figure 3B). Solid horizontal line shows the average, dotted lines show two standard deviations. (B) Cumulative counts of differentially expressed genes between YrDNA-gross and wild-type Y (at P<0.01) in males (black), females (gray), and females multiplied by a correction (dotted gray) to more easily compare trends (data from Figure 3C). (0.29 MB TIF) [file pgen.1001376.s006.tif]

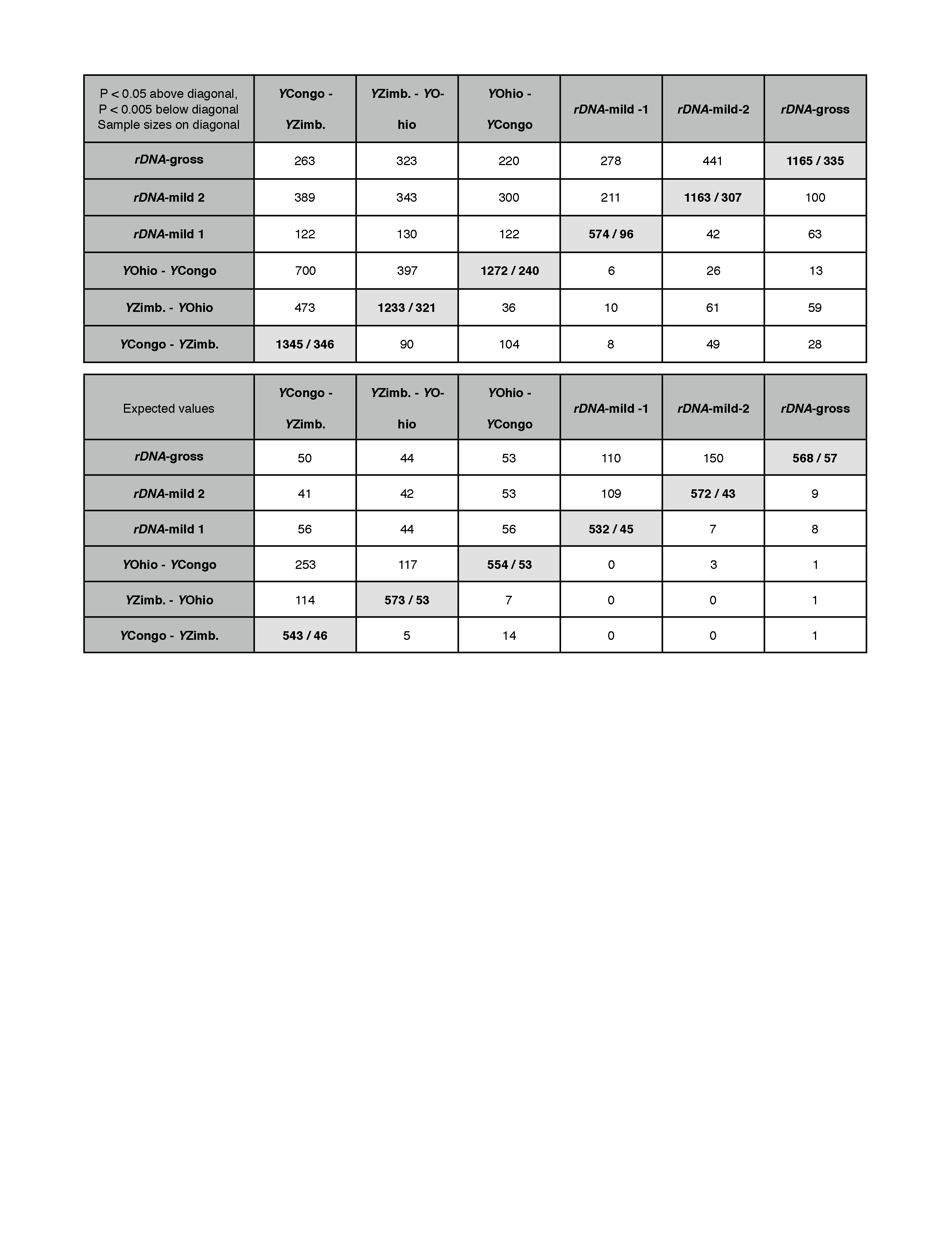

Supplement: Figure S7 — (A) Observed number of shared differentially expressed genes across pairwise chromosome comparisons. P<0.05 data are shown above the diagonal, P<0.005 are shown below the diagonal, and total number (shared plus unique) of differentially expressed genes (P<0.05/P<0.005) are shown on the diagonal (bold, gray background). (B) Expected numbers of genes shared between induced rDNA deletion Y chromosomes and natural Y chromosomes, calculated from randomized datasets (N for individual pairwise comparisons are on diagonal) at P<0.05 (above diagonal) and P<0.005 (below diagonal). (0.27 MB TIF) [file pgen.1001376.s007.tif]
